# Posttraumatic urethral hemorrhage associated with an arteriospongious fistula and managed with catheter-directed embolization: a report of 2 cases

**DOI:** 10.1186/s42155-025-00593-4

**Published:** 2025-10-02

**Authors:** Stijn De Bondt, Steven Joniau, Maarten Albersen, Geert Maleux

**Affiliations:** 1 Department of Radiology, University Hospitals KU Leuven, Louvain, Belgium; 2 Department of Imaging & Pathology, KU Leuven, Louvain, Belgium; 3 Department of Urology, University Hospitals KU Leuven, Louvain, Belgium

**Keywords:** Trauma, Urethral hemorrhage, Embolization

## Abstract

**Background:**

Urethral bleeding can be related to iatrogenic and non-iatrogenic trauma; selective internal iliac angiography may identify contrast extravasation with or without a pseudoaneurysm at the level of the distal internal pudendal or bulbourethral artery. Here, we describe another, yet unreported vascular lesion of the bulbourethral artery related to urethral injury.

**Case presentation:**

Two patients with a iatrogenic and non-iatrogenic urethral bleeding respectively are presented. Conservative management, including Foley catheter placement and endoscopic management were unsuccessful. Selective internal pudendal angiography revealed an arteriospongious fistula without clear contrast extravasation into the urethral lumen; super-selective embolization with microcoils and non-adhesive liquid embolics was safely performed and successfully stopped the bleeding. The postinterventional course was uneventful and both patients recovered without sequelae.

**Conclusions:**

Traumatic urethral bleeding might be related to an arteriospongious fistula which can be successfully managed with super-selective coil and liquid embolic embolization.

**Graphical Abstract:**

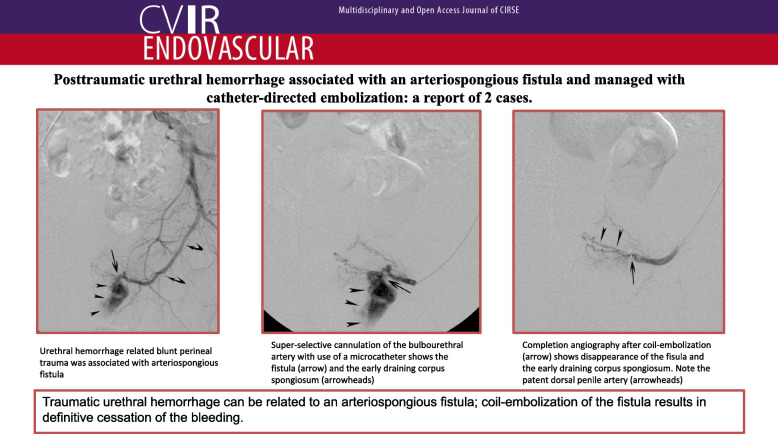

## Introduction

Urethral bleeding related to iatrogenic or non-iatrogenic trauma is a rare clinical condition which is traditionally managed with conservative methods, including endoscopic clot removal, placement of an indwelling Foley catheter for some weeks and administration of blood products if needed [1]. If the bleeding persists despite optimal conservative management, endovascular or surgical intervention is mandatory [1]. Today, in many centers the endovascular approach is the first choice. In this report we present two rare cases of persistent traumatic urethral hemorrhage related to an arteriospongious fistula and treated with microcoil embolization after failed conservative management.

## Case report 1

**Fig. 1:**
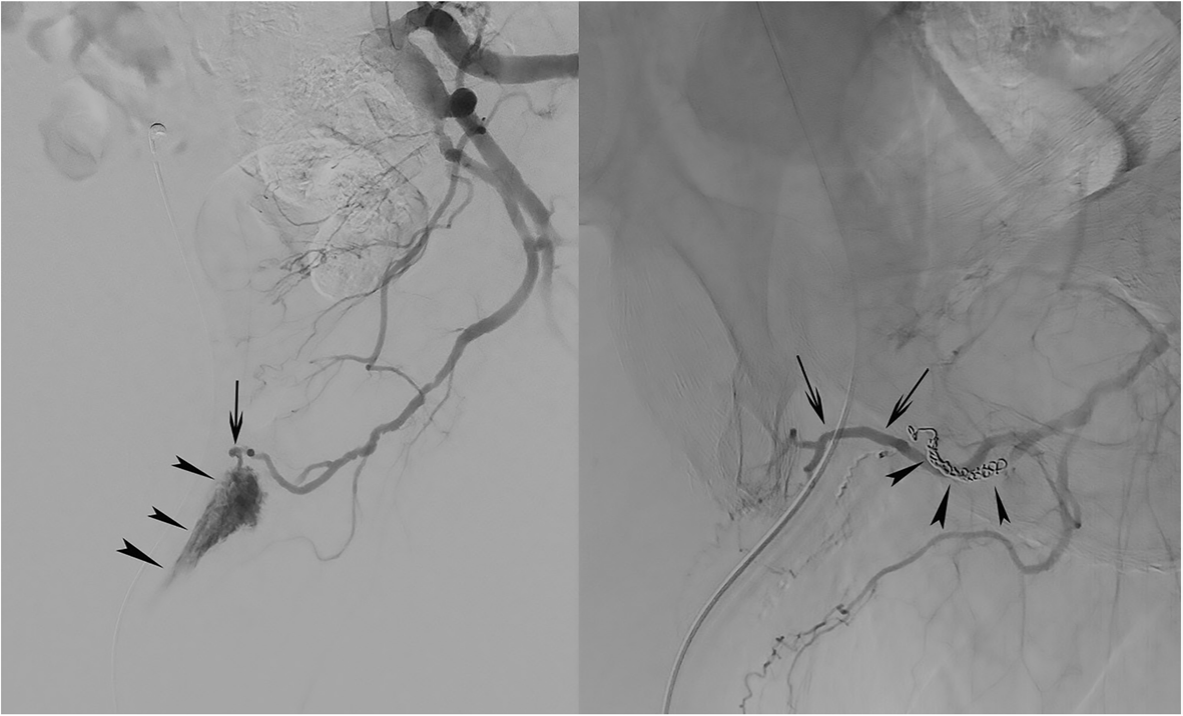
**a** Selective left internal iliac artery angiography. Contrast injection revealed a direct fistulous communication between the bulbourethral artery (arrow) and the corpus spongiosum (arrowheads). **b** Completion selective angiography after coil (arrowheads) and Onyx embolization. Contrast injection shows the disappearance of the arteriospongious fistula and a patent dorsal penile artery (arrows)

A 74-year-old man underwent a laparoscopic microwave ablation for a focal, 3 cm diameter hepatocellular carcinoma in segment 8 of the right liver lobe. Postoperatively, the patient presented with acute urinary retention necessitating placement of a Foley catheter. However, catheter insertion was difficult and resulted in traumatic urethral hemorrhage; there was no previous history of lower urinary tract symptoms. Cystoscopic clot removal temporarily controlled the bleeding; however, one week later, the patient presented with recurrent urethral hemorrhage and a drop in Hemoglobin (10.4 g/dL). He was referred to the interventional radiology unit for angiographic diagnosis and definitive endovascular management. After counseling the patient and obtaining informed consent, a 4 French (F) vascular sheath was placed, under local anesthesia, in the right common femoral artery. The left internal iliac artery was cannulated with use of a 4 F Cobra catheter (Glide cath, Terumo Europe, Leuven, Belgium) and selective angiography revealed an arteriospongious fistula between the bulbourethral artery and the corpus spongiosum without visualization of any additional contrast extravasation or pseudoaneurysm (Fig. 1a). Through a microcatheter (Progreat 2.4, Terumo Europe, Leuven, Belgium), 2 microcoils (4 × 4 Target microcoil, Boston Scientific, Natick, MA, USA) and 0.4 ml of ethylene vinyl alcohol (EVOH)-based liquid (Onyx 34, Medtronic, Minneapolis, MN, USA) were successfully placed in the proximal part of the left bulbourethral artery, resulting in complete occlusion of the right-sided fistula (Fig. 1b). Further, selective angiography of the contralateral, right internal iliac artery also revealed a small fistula between the right bulbourethral artery and the corpus cavernosum which was subsequently and successfully occluded with the same type of Target microcoils and Onyx-34. Completion selective angiography of both right and left internal iliac artery showed complete closure of the fistula and a patent, bilateral dorsal penile artery. Clinically, the macroscopic hematuria resolved and two days later the patient was discharged with a Foley catheter in situ. Two weeks later, the catheter was removed in the outpatient clinic; no recurrent hematuria, neither other urinary problems were reported by the patient.

## Case report 2

**Fig. 2:**
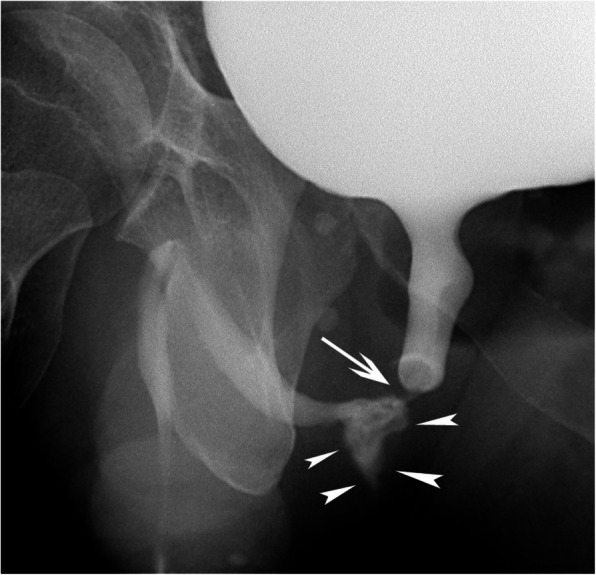
Retrograde urethrography. Oblique plain film after retrograde contrast injection clearly demonstrates the post-traumatic stricture (arrow) and the contrast extravasation (arrowheads) at the posterior part of the pars bulbosa urethrae

**Fig. 3:**
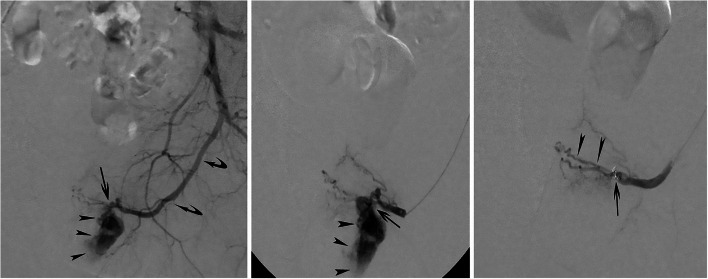
**a** Selective, left internal iliac angiography. **b** Super-selective angiography of the left internal pudendal artery. Contrast injection reveals the feeding, internal pudendal artery main branch (curved arrows), the arteriospongious fistula (arrow) and early drainage of contrast medium into the corpora spongiosa (arrowheads). **c** Completion angiography after coil-embolization (arrow). Contrast injection shows absence of opacification of the fistula to corpus spongiosum and remained patency of the dorsal penile artery (arrowheads)

A 46-year-old man presented at the emergency department with a meatal bleeding, painful urinary retention and perineal subcutaneous hematoma after blunt perineal trauma. Initial management included placement of a suprapubic catheter, intravenous oral antibiotics and pain management. However, during the next 4 weeks, the patient presented with recurrent episodes of macroscopic hematuria and a cystoscopy was performed for clot removal and an attempt was made to endoscopically close the urethral tear. Another four weeks later, the patient was re-admitted for retrograde urethrography, cystoscopic clot removal and Foley catheter insertion. Urethrography revealed some contrast extravasation at the posterior part of the pars bulbosa urethrae associated with a clear stricture (Fig. 2); this stricture was resolved by a cystoscopic Sachse urethrotomy. After repeat clot removal, recurrent episodes of macroscopic hematuria still occurred during the following weeks; finally, the patient was referred to the interventional radiology unit for endovascular management. After counseling the patient and obtaining informed consent, a 4 French (F) vascular sheath was placed, under local anesthesia, in the right common femoral artery. Both internal iliac arteries were cannulated with use of a 4 F Cobra catheter (Glide Cath, Terumo Europe, Leuven, Belgium) revealing a left-sided arteriospongious fistula between the bulbourethral artery and the corpus spongiosum without visualization of any additional contrast extravasation or pseudoaneurysm (Fig. 3a,b); at the right side no injured vessel was identified. Super-selective embolisation of the fistula was performed through a microcatheter (Progreat 2.4, Terumo Europe, Leuven, Belgium) with use of 2 microcoils (4 × 4 Target microcoil, Boston Scientific, Natick, MA, USA). Completion internal iliac artery angiography revealed complete occlusion of the arteriospongious fistula, without need for additional liquid embolic embolization, and remained patency of the dorsal penile artery (Fig. 3c).

Three weeks later, the Foley catheter was definitively removed and no recurrent hematuria was reported.

## Discussion

The two presented cases are dealing with intermittent urethral bleeding related to traumatic arteriospongious and arteriourethral fistula despite the absence of urethral luminal contrast extravasation on selective angiographies. From an anatomical perspective, the bulbourethral artery, the corpus spongiosum and the anterior part of the urethra are in close contact within the ventral part of the penis [2]; blunt and/or penetrating, local trauma subsequently can lead to a fistula between these anatomic structures. In most case reports on traumatic urethral hemorrhage, however, angiographic evaluation demonstrates a uni- or bilateral pseudo-aneurysm of the distal part of the pudendal artery main branch [3, 4] or the bulbourethral artery [5–9] with [3, 8] or without contrast extravasation into the urethral lumen [5–9]. From a clinical perspective, an arteriospongious fistula is not associated with clinical signs of high-flow priapism as demonstrated in the two presented cases. Adversely, when an arteriourethral fistula is associated with an arteriocavernosal fistula, posttraumatic urethral hemorrhage can be associated with symptoms of high-flow priapism as demonstrated by Kondo et al. [4]. Last, Vihtelic et al. recently published a case of urethral trauma associated with meatal bleeding and erectile dysfunction which completely resolved after super-selective coil-embolization [10]. In the two presented cases, no clinical evidence of erectile dysfunction was found.

Finally, super-selective embolization using microcoils seems to be the treatment of choice in these patients [3, 8, 9]: cessation of the bleeding is demonstrated in nearly all cases and no adverse events, including penile ischemia or erectile dysfunction are reported, even in cases of bilateral, super-selective embolization, potentially related to the rich, penile collateral network. If completion angiography after microcoil embolization still demonstrates persistent flow through the fistula, additional viscous EVOH-based liquid embolics might be needed to completely occlude the fistula; viscous, EVOH-based liquid embolics might be preferred to glue related to the lower risk for non-target embolization, both antegradely in the corpora spongiosa as well as retrogradely into the dorsal penile artery.

## Conclusion

Two cases of posttraumatic urethral hemorrhage associated with an arteriospongious fistula are reported. Definitive diagnosis was made, based on superselective catheter-angiography and definitive treatment was made with use of microcoils with or without additional EVOH-based liquid embolics.

## Data Availability

The datasets used and/or analysed during the current study are available from the corresponding author on reasonable request.

## Abbreviation

F: French
